# Syphilitic Interstitial Gingivitis

**Published:** 1903-02

**Authors:** Eugene S. Talbot

**Affiliations:** Chicago


### SYPHILITIC INTERSTITIAL GINGIVITIS.

BY EUGENE S. TALBOT, M.D., D.D.S., CHICAGO.[1]

In the past year three patients with syphilitic infection of the mouth have been referred to me. A young Swedish domestic, twenty-four years of age, contracted the disease by kissing. Syphilitic patches appeared upon the lower lip the size of a large filbert, involving the gums and alveolar process, with considerable uneasiness and pain at the roots of the teeth. The ulcer was nearly healed when she came to me.

Mr. H., thirty-two years of age, also contracted the disease by kissing. The central lesion appeared at the lower lip. When I saw him secondary syphilis had caused his body to become completely covered with a rash. Large mucous patches covered the mouth. The gums and alveolar processes were of a deep-red color and the teeth were sore to touch.

Mr. A., twenty-seven years of age, clerk in a wholesale dry-goods house, became infected by moistening labels with saliva. Although under treatment, his face and body became completely covered with eruptions and the alveolar process and gums were badly inflamed. The cervical glands in all cases were enlarged.

These cases are receiving special local treatment by me. This consists in cleansing the teeth (a special set of instruments are kept for this purpose) and the local treatment of iodine. Mrs. H., the case I reported in the International Dental Journal some time ago, returned with slight uneasiness in the alveolar process at the third right inferior molar. The usual iodine treatment is being given. It would seem, from the experience I have had with local iodine treatment in syphilitic interstitial gingivitis, that though relief from uneasiness and pain is all that one could wish, it is apt to return unless systemic treatment is adopted.

The uneasiness and pain in the alveolar process about the roots of the teeth is not confined to syphilitic interstitial gingivitis alone.

---

[1] Fellow of the Chicago Academy of Medicine.

Interstitial gingivitis from whatever cause will produce the same symptoms, including redness, due to the inflammatory process and bone absorption extending throughout the alveolar process.

In the lower animals this uneasiness and pain is best illustrated by the cribbing of the horse returned to the stall after a summer's outing.

